# *SMAD7* polymorphisms and colorectal cancer risk: a meta-analysis of case-control studies

**DOI:** 10.18632/oncotarget.12285

**Published:** 2016-09-27

**Authors:** Yongsheng Huang, Wenting Wu, Meng Nie, Chuang Li, Lin Wang

**Affiliations:** ^1^ Institute of Basic Medical Sciences and School of Basic Medicine, Chinese Academy of Medical Sciences and Peking Union Medical College, Beijing 100730, China; ^2^ Department of Epidemiology, Richard M. Fairbanks School of Public Health, Melvin & Bren Simon Cancer Center, Indiana University, Indianapolis, IN 46202, USA

**Keywords:** SMAD7, colorectal cancer, polymorphism, meta-analysis

## Abstract

Mothers against decapentaplegic homolog 7 (*SMAD7*) inhibits the transforming growth factor-β (TGF-β) signaling pathway, which regulates carcinogenesis and cancer progression. A number of studies have reported that *SMAD7* polymorphisms (rs4464148, rs4939827, and rs12953717) are associated with colorectal cancer (CRC) risk, but the results from these studies remain conflicting. To determine a more precise estimation of the relationship between *SMAD7* and CRC, we undertook a large-scale meta-analysis of 63 studies, which included a total of 187,181 subjects (86,585 cases and 100,596 controls). The results of our meta-analysis revealed that the C allele of rs4464148 [CC vs. TT+TC, odds ratio (OR) =1.23, 95% confidence interval (CI): 1.14–1.33, *P* < 0.01], the T allele of rs4939827 [TT vs. CC+TC, odds ratio OR=1.15, 95%CI:1.07–1.22, *P* < 0.01] and the T allele of rs12953717 [TT vs. CC+TC, OR =1.22, 95%CI:1.16–1.29, *P* < 0.01] were all associated with the increased CRC risk. Subgroup analysis according to ethnicity showed rs4464148 and rs12953717 were associated with the risk of CRC in both Caucasians and Asians, whereas rs4939827 was a risk polymorphism for CRC specifically in Caucasians. In summary, this large-scale meta-analysis indicated that *SMAD7* polymorphisms (rs4464148, rs4939827, and rs12953717) correlate with CRC.

## INTRODUCTION

Cancer is caused by the dysfunction of intricate signaling pathways, leading to abnormal growth, metastasis, and many other events [1]. The transforming growth factor β (TGF-β) signaling pathway is one of major tumor-regulatory pathways, exerting critical tumor-suppressive functions in the early stages of tumorigenesis [2, 3]. When TGF-β signaling is activated, downstream SMAD2 and SMAD3 proteins are phosphorylated, forming a complex with SMAD4 and then translocating to the nucleus to turn on and off the transcription of a wide range of target genes [4, 5]. *SMAD7* inhibits TGF-β signaling by preventing the formation of the SMAD2/SMAD4 complex [6]. It also interacts with activated TGF-β type I receptor and blocks the phosphorylation and activation of SMAD2 [6].

*SMAD7* has also been reported to affect tumorigenesis via several other mechanisms. First, in FET-1 colon cancer cells, *SMAD7* induces the expression of I*κ*B, thereby repressing NF-*κ*B activity [7]. Secondly, *SMAD7* up-regulates MYC expression and WNT signaling via interactions with β-catenin in breast cancer [8] and hepatocellular carcinoma [9]. In addition, *SMAD7* inhibits ERK1/2, JNK1/2, and p38 MAPKs under some circumstances related with tumorigenesis, such as erythroid differentiation [10] and chondrocyte differentiation [11].

In 2007, Broderick and co-workers [12] conducted a genome-wide association study and identified three polymorphic variants in intron 3 of *SMAD7* (rs4464148, rs4939827, and rs12953717). Furthermore, they found these *SMAD7* polymorphisms were associated with CRC adenomas and carcinomas [12]. In a number of other studies these *SMAD7* polymorphisms have been associated with the risk of developing multiple cancers, including CRC [12–14], renal [15], and liver cancer [16]. However, other case-control studies have reported that these polymorphisms are not associated with cancer risk, in CRC [17–19], breast cancer [20], and lymphocytic leukemia [21]. These inconsistencies may be partially due to the relatively small sample sizes in each of these studies. Therefore, we performed a large-scale meta-analysis of all eligible published studies to derive a more precise quantitative assessment of the association between *SMAD7* polymorphisms and CRC risk.

## RESULTS

### Study selection and characteristics

**Figure 1:**
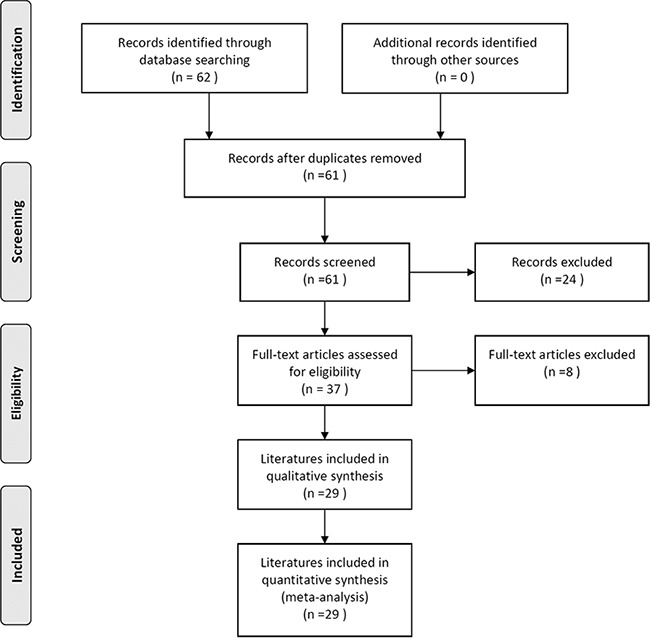
Flowchart of the literature selection process

Figure 1 is a flowchart explaining the study selection process. A total of 62 articles were initially retrieved from PubMed, Web of Science, EBSCO, and Embase electronic databases (last updated in June, 2016). Based on the search criteria, we excluded 33 ineligible records after carefully reviewing the full text and data, leaving 29 articles published between 2007 and 2016 for our quantitative meta-analysis.

The characteristics of *SMAD7* polymorphisms (rs4464148, rs4939827, and rs12953717) in selected studies are shown in Table 1. There were 64 eligible studies from 29 articles analyzing the relationship of *SMAD7* polymorphisms and CRC risk. Among these studies, one was conducted on rs12953717, with a relatively small sample size (308 subjects) [22], which seems to have affected the results dramatically. Therefore, this study was excluded from analysis. Finally, 63 studies (published from 2007-2016) including 187,181 subjects (86,585 cases and 100,596 controls) were used to estimate the risk of developing CRC with *SMAD7* polymorphisms. Each subpopulation in the literature was treated as a separate study in our meta-analysis. Populations were divided into ethnic categories. The Newcastle-Ottawa Scale (NOS) was used for quality assessment [23] and all of the studies achieved moderately high quality scores above 6 (Table 1). Among the included studies, 12 were conducted on rs4464148 (18,303 cases and 16,964 controls), 37 on rs4939827 (48,751 cases and 61,529 controls), and 14 on rs12953717 (19,531 cases and 22,103 controls).

**Table 1:** Main characteristics of all case-control studies included in the meta-analysis

SNP	Author	Year	Ethnicity	Cancer type	Case			Control			HWE (Control *P* value)	Study design	Genotyping method	Quality assessment
**rs4464148**					**TT**	**TC**	**CC**	**TT**	**TC**	**CC**				
	Broderick et al. [12] -A group	2007	Caucasian	Colon	389	425	116	486	394	80	0.991	GWAS	Illumina	8
	-B group	2007	Caucasian	Colon	2017	1952	472	1886	1617	346	0.982	Replication	Allele-PCR	8
	-C group	2007	Caucasian	Colon	922	845	193	827	696	146	0.980	Replication	Allele-PCR	8
	-D group	2007	Caucasian	Colon	422	408	99	171	137	27	0.952	Replication	Allele-PCR	8
	Thompson et al. [28]	2009	Caucasian	Colon	269	231	61	342	324	53	0.045	Replication	TaqMan	8
	Curtin et al. [43]	2009	Caucasian	Colon	503	472	95	535	423	89	0.678	Replication	SNPlex	8
	Pittman et al. [44]	2009	Caucasian	Colon	1161	1107	264	1095	1277	235	0.996	Replication	Allele-PCR	8
	Ho et al. [35]	2011	Asian	Colon	739	146	7	770	116	4	0.869	Replication	Sequenom	7
	Zhang et al. [38]	2014	Asian	Colon	1	52	675	14	305	2957	0.999	Replication	TaqMan	8
	Kurlapska et al. [17]	2014	Caucasian	Colon	1214	1228	400	84	96	33	0.523	Replication	Sequenom	7
	Damavand et al. [29]	2015	Caucasian	Colon	138	78	37	113	101	20	0.700	Replication	Taqman	7
	Serrano-Fernandez et al. [45]	2015	Caucasian	Colon	507	517	141	561	490	114	0.643	Replication	Taqman	8
**rs4939827**					**CC**	**TC**	**TT**	**CC**	**TC**	**TT**				
	Broderick et al. [12] -A group	2007	Caucasian	Colon	153	449	328	229	480	251	0.987	GWAS	Illumina	8
	-B group	2007	Caucasian	Colon	852	2178	1392	845	1915	1084	0.989	Replication	Allele-PCR	8
	-C group	2007	Caucasian	Colon	387	982	623	410	840	430	0.995	Replication	Allele-PCR	8
	-D group	2007	Caucasian	Colon	194	477	292	76	171	96	0.923	Replication	Allele-PCR	8
	Tenesa et al. [14] -Scotland(GWAS)	2008	Caucasian	Colon	538	1521	926	706	1508	845	0.506	GWAS	Illumina	8
	-Japan	2008	Asian	Colon	233	1582	2576	131	1028	2019	0.992	Replication	TaqMan	8
	-Canada	2008	Caucasian	Colon	225	593	355	284	576	322	0.402	Replication	TaqMan	8
	-England	2008	Caucasian	Colon	418	1120	694	546	1126	578	0.959	Replication	TaqMan	8
	-Spain	2008	Caucasian	Colon	62	156	131	57	143	95	0,808	Replication	TaqMan	8
	-Germany	2008	Caucasian	Colon	420	1071	659	541	1057	530	0.765	Replication	TaqMan-	8
	-Germany	2008	Caucasian	Colon	289	617	412	378	704	358	0.403	Replication	TaqMan	8
	-Scotland	2008	Caucasian	Colon	156	420	254	189	446	288	0.497	Replication	TaqMan	8
	-Israel	2008	Caucasian	Colon	267	638	447	312	627	397	0.035	Replication	TaqMan	8
	Curtin et al. [43]	2009	Caucasian	Colon	221	520	324	229	538	274	0.251	Replication	SNPlex	8
	Thompson et al. [28]	2009	Caucasian	Colon	125	275	154	146	378	185	0.064	Replication	TaqMan	8
	Pittman et al. [44]	2009	Caucasian	Colon	785	1250	497	725	1300	582	0.987	Replication	Allele-PCR	8
	Slattery et al. [46]	2010	Caucasian	Colon	360	773	457	492	992	503	0.947	Replication	TaqMan	8
	Xiong et al. [33]	2010	Asian	Colon	1370	677	77	1442	570	74	0.061	Replication	T-ARMS-PCR	8
	von Hoslt et al. [47]	2010	Caucasian	Colon	395	886	501	387	884	408	0.930	Replication	deCode test	8
	Kupfer et al. [48]	2010	African	Colon	379	340	76	455	429	101	0.994	Replication	Sequenom	7
			Caucasian	Colon	88	199	112	85	183	99	0.981	Replication	Sequenom	7
	Mates et al. [49]	2010	Caucasian	Colon	28	37	27	15	57	23	0.061	Replication	Centaurus	6
	Mates et al. [50]	2011	Caucasian	Colon	42	69	42	32	106	43	0.225	Replication	Centaurus	7
	Cui et al. [34]	2011	Asian	Colon	1628	1007	155	2247	1190	147	0.501	Replication	Illumina	8
	Li et al. [22]	2011	Asian	Colon	73	53	12	81	73	14	0.665	Replication	Sequenom	7
	Ho et al. [35]	2011	Asian	Colon	343	420	129	376	405	109	0.997	Replication	Sequenom	7
	Song et al. [36]	2012	Asian	Colon	399	232	10	732	272	33	0.214	Replication	TaqMan	6
	Lubbe et al. [51]	2012	Caucasian	Colon	444	969	624	1394	3021	1636	0.993	Replication	Allele-PCR	7
	Garcia-Albeniz et al. [52]	2012	Caucasian	Colon	90	233	118	538	1120	600	0.731	Replication	TaqMan	8
	Phipps et al. [53]	2012	Caucasian	Colon	657	1526	884	574	1597	1112	0.988	Replication	TaqMan	7
	Kirac et al. [54]	2013	Caucasian	Colon	63	143	96	172	291	131	0.705	Replication	Illumina	8
	Yang et al. [37]	2014	Asian	Colon	342	298	65	891	752	159	0.985	Replication	Allele-PCR	7
	Kurlapska et al. [17]	2014	Caucasian	Colon	54	93	65	716	1394	730	0.330	Replication	Sequenom	7
	Zhang et al. [38]	2014	Asian	Colon	400	277	51	1894	1170	212	0.858	Replication	TaqMan	7
	Hong et al. [19]	2015	Asian	Colon	126	63	9	182	127	19	0.608	Replication	Illumina	7
	Baert-Desurmont et al. [55]	2016	Caucasian	Colon	89	157	104	191	493	343	0.555	Replication	SNaPshot	8
	Abd EI-Fattah et al. [18]	2016	Caucasian	Colon	20	35	22	11	15	10	0.319	Replication	TaqMan	7
**rs12953717**					**CC**	**TC**	**TT**	**CC**	**TC**	**TT**				
	Broderick et al.-A group [12]	2007	Caucasian	Colon	159	309	151	326	467	167	0.991	GWAS	Illumina	8
	-B group	2007	Caucasian	Colon	1247	2204	973	1248	1898	722	0.994	Replication	Allele-PCR	8
	-C group	2007	Caucasian	Colon	582	991	422	558	834	312	0.990	Replication	Allele-PCR	8
	-D group	2007	Caucasian	Colon	277	468	198	106	168	67	0.976	Replication	Allele-PCR	8
	Middeldorp et al. [13]	2009	Caucasian	Colon	301	493	201	482	643	215	0.982	Replication	TaqMan	7
	Curtin et al. [43]	2009	Caucasian	Colon	314	530	226	332	521	188	0.509	Replication	SNPlex	8
	Thompson et al. [28]	2009	Mixed	Colon	196	248	116	220	370	129	0.218	Replication	TaqMan	8
	Pittman et al. [56]	2009	Caucasian	Colon	716	1261	555	859	1275	473	0.998	Replication	Allele-PCR	8
	Kupfer et al. [48]	2010	African	Colon	401	327	67	525	388	72	0.979	Replication	Sequenom	7
		2010	Caucasian	Colon	197	121	81	119	180	68	0.996	Replication	Sequenom	7
	Slattery et al. [46]	2010	Caucasian	Colon	503	754	332	676	928	327	0.779	Replication	Illumina	8
	Ho et al. [35]	2011	Asian	Colon	276	343	97	304	345	65	0.557	Replication	Sequenom	7
	Scollen et al. [56]	2011	Mixed	Colon	710	1031	425	730	1083	437	0.326	Replication	TaqMan	8
	Zhang et al. [38]	2014	Asian	Colon	418	263	47	1947	1135	194	0.096	Replication	TaqMan	8

**SNP**: single nucleotide polymorphisms: **HWE**: Hardy-Weinberg equilibrium.

### Quantitative data synthesis

#### *SMAD7* rs4464148 polymorphism

For each study, we investigated the association between the *SMAD7* rs4464148 polymorphism and CRC risk, assuming different inheritance models. When all eligible studies were pooled into the meta-analysis, significant associations were found for the recessive genetic model (Table 2): CC vs. TC+TT (OR = 1.23; 95% CI: 1.14–1.33; *P_Z_* < 0.01; *P_H_* = 0.43], while only a slight association was found for the dominant genetic model: CC +TC vs. TT (OR = 1.10; 95% CI: 0.99–1.22; *P_Z_* = 0.51; *P_H_* = 0.00). Subgroup analysis according to ethnicity showed that rs4464148 was significantly associated with CRC risk in both Caucasian and Asian populations (Table 2).

**Table 2:** Meta-analysis of the association between *SMAD7* polymorphisms and colorectal cancer risk

SNP	Comparison	Subgroup	Heterogeneity test		Model	*P_Z_*	*P_E_*	OR (95% CI)
			*I^2^* (%)	*P_H_*				
rs4464148	CC *vs*. TT+TC	Overall	1.3	0.43	F	<0.01	0.13	1.23(1.14–1.33)
		Caucasian	12.3	0.33	F	<0.01		1.22(1.13–1.32)
		Asian	0	0.71	F	0.03		1.39(1.04–1.87)
	CC+TC *vs.* TT	Overall	73.8	0.00	R	0.07	0.51	1.10(0.99–1.22)
		Caucasian	76.8	0.00	R	0.16		1.08(0.97–1.21)
		Asian	0	0.41	F	0.02		1.36(1.05–1.75)
	C *vs*. T	Overall	66.2	0.00	R	<0.01	0.36	1.12(1.04–1.19)
		Caucasian	67.7	0.00	R	0.01		1.10(1.02–1.18)
		Asian	66	0.09	F	<0.01		1.35(1.12–1.63)
rs4939827	TT *vs*. CC+TC	Overall	73.3	0.00	R	<0.01	0.89	1.15(1.07–1.22)
		Caucasian	61.2	0.00	R	<0.01		1.19(1.12–1.26)
		Asian	75.8	0.00	R	0.73		1.04(0.84–1.28)
	TT+TC *vs*. CC	Overall	71.8	0.00	R	<0.01	0.14	1.13(1.07–1.20)
		Caucasian	71.6	0.00	R	<0.01		1.16(1.08–1.24)
		Asian	74.0	0.00	R	0.31		1.07(0.94–1.23)
	T *vs*. C	Overall	79.6	0.00	R	<0.01	0.45	1.11(1.06–1.16)
		Caucasian	74.7	0.00	R	<0.01		1.13(1.08–1.18)
		Asian	56.9	0.00	R	0.33		1.07(0.94–1.21)
rs12952717	TT *vs*. CC+TC	Overall	13.2	0.31	F	<0.01	0.54	1.22(1.16–1.29)
		Caucasian	0	0.87	F	<0.01		1.25(1.18–1.32)
		Asian	54.9	0.14	F	0.02		1.31(1.04–1.65)
	TT+TC *vs*. CC	Overall	51.3	0.02	R	<0.01	0.66	1.15(1.08–1.23)
		Caucasian	45.3	0.06	F	<0.01		1.19(1.13–1.25)
		Asian	0.0	0.54	F	0.082		1.12(0.99–1.28)
	T *vs*. C	Overall	51.5	0.02	R	<0.01	0.85	1.13(1.09–1.19)
		Caucasian	29.8	0.17	F	<0.01		1.16(1.12–1.20)
		Asian	19.6	0.27	F	0.02		1.13(1.02–1.25)

*****P**_H_*** : *P* value of heterogeneity test; *****P**_Z_*** : *P* value of Z test; *****P**_E_*** : *P* value of Egger's test. R: random-effects model. F: fixed-effects model

#### *SMAD7* rs4939827 polymorphism

**Figure 2:**
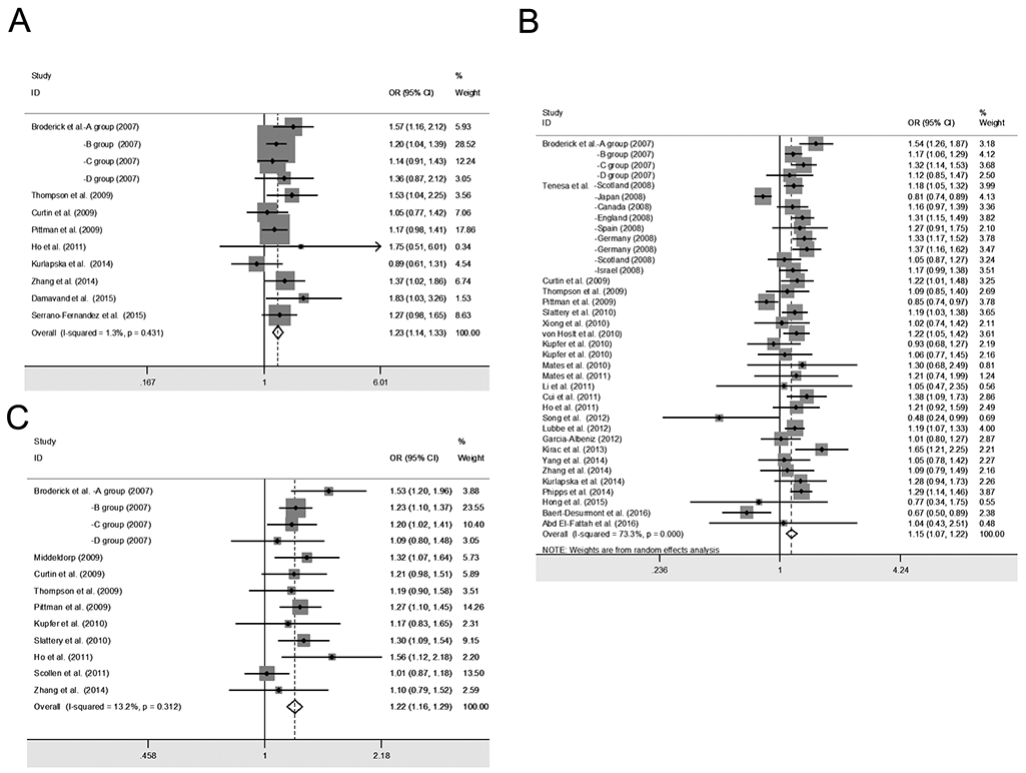
Forest plot of cancer risk associated with the *SMAD7* polymorphisms in colorectal cancer studies with recessive genetic models The squares and horizontal lines correspond to the study-specific odds ratio (OR) and 95% confidence interval (95% CI). The area of the squares reflects the weight (inverse of the variance). **A.** *SMAD7* rs4464148; **B.** *SMAD7* rs4939827; **C.** *SMAD7* rs12953717.

Similarly, we investigated the association between the *SMAD7* rs4939827 polymorphism and CRC risk. Significant associations were found for both the recessive (Figure 2): TT vs. TC+CC (OR = 1.15; 95% CI: 1.07–1.22; *P_Z_* < 0.01; *P_H_* = 0.00) and the dominant genetic models: TT+ TC vs. CC (OR = 1.13; 95% CI: 1.07–1.20; *P_Z_* < 0.01; *P_H_* = 0.00; Table 2). Subgroup analysis according to ethnicity showed that rs4939837 was significantly associated with CRC risk in the Caucasian population (27 studies: 36,062 cases and 43,518 controls): TT vs. TC+CC (OR = 1.19; 95% CI: 1.12–1.26; *P_Z_* < 0.01; *P_H_* = 0.00 for heterogeneity), whereas it had no association with CRC risk among Asians (9 studies: 12,607 cases and 16,349 controls): TT vs. TC+CC (OR = 1.04; 95% CI: 0.84–1.28; *P_Z_* = 0.73; *P_H_* = 0.00; Table 2).

#### *SMAD7* rs12953717 polymorphism

In this meta-analysis, a strong association between the rs12953717 polymorphism and CRC risk was found for both the recessive: TT vs. CC+TC (OR = 1.22; 95% CI: 1.16–1.29; *P_Z_* < 0.01; *P_H_* = 0.31) and the dominant genetic models: TT+TC vs. CC (OR = 1.15; 95% CI: 1.08–1.23; *P_Z_* < 0.01; *P_H_* = 0.02; Table 2). Further subgroup analysis based on ethnicity showed that rs12953717 was significantly associated with the risk of CRC in both Caucasians and Asians (Table 2).

### Sensitivity analyses and publication bias

Our results suggested that the influence of individual data sets to the pooled ORs were not significant. Sensitivity analysis showed that no single study qualitatively altered the pooled ORs, providing evidence of the stability of the meta-analysis (Supplementary Figure S1). Funnel plots and Egger's test were performed to assess publication bias. The results suggested that there was no publication bias for the comparison of rs4464148 allele C vs. allele T (*t* =0.96, *P_E_* = 0.36), rs4939827 allele T vs. allele C (*t* =−0.76, *P_E_* = 0.45), or rs12953717 allele T vs. allele C (*t* =−0.19, *P_E_* = 0.85). The shape of Begg's funnel plot did not reveal any obvious asymmetry (Supplementary Figure S2).

## DISCUSSION

TGF-β signaling is essential for maintaining homeostasis, cell differentiation, and tumor suppression [3, 24, 25]. Increased production of TGF-β occurs in various tumor types, such as CRC [26]. As one of the key effectors of TGF-β signaling, perturbation of *SMAD7* expression has been documented to influence CRC progression [7][27]. Though the functional role of the *SMAD7* polymorphisms (rs4464148, rs4939827, and rs12953717) has not yet been interpreted, a number of published epidemiological studies have reported that these polymorphisms are correlated with the risk of developing multiple cancers [12, 28, 29]. However, other studies have reported that these polymorphisms are not associated with cancer development [17–20].

These conflicting studies based their conclusions on small numbers of samples and different detection methods. Therefore ameta-analysis from large-scale samples of all available studies is required to have a more accurate assessment as to whether the *SMAD7* polymorphisms are related to risk of developing CRC. Our group has already used meta-analysis to systematically investigate the association between cancer risk and several SNPs involved in TGF-β signaling [30–32]. In this meta-analysis, we found *SMAD7* polymorphisms (rs4464148, rs4939827, and rs12953717) in the combined population were all significantly associated with CRC risk. Subgroup analysis according to ethnicity showed that rs4464148 and rs12953717 were significantly associated with the risk of CRC among both Caucasian and Asian population, whereas rs4939827 seems to be a risk polymorphism for CRC only within a Caucasian population. There could be several possibilities to explain such a differential association. First, the difference in association may result from differences in socioeconomic environment, regional dietary habits, and race. Second, the number of rs4939827 in Asian studies is still not as large as desired. In addition, the results from nine studies incorporated in this meta-analysis conflict with each other [14, 19, 22, 33–38]. Therefore, more Asian studies are still needed to clearly evaluate the interactions of *SMAD7* rs4939827 and CRC in this ethnic group.

One recent study [39] also assessed the associations between these three SNPs and CRC risk by meta-analysis; however, there were significant limitations. First, the number of studies included in their analysis was smaller than ours. Only 4 publications for rs4464148, which also lack relevant studies for Asian population, and 13 publications for rs4939827 were included in their meta-analysis, while 9 publications for rs4464148 and 25 publications for rs4939827 were included in our work. Second, they only analyzed the relationship between *SMAD7* polymorphisms and CRC risk under an allelic model, while we also analyzed under dominant and recessive models. Therefore, our updated meta-analysis at a much larger scale clearly provides a more credible and reliable assessment for the association between *SMAD7* polymorphisms and the risk to develop CRC.

Nonetheless, we also wish to acknowledge the limitations in our study. First, we stratified the studies by ethnic subtypes as Caucasian and Asian. However, we could not assess the association in the African population due to the insufficient number of African studies. Second, further subtle adjusted analysis could be carried out if more detailed individual information was available. Third, we only assessed the association of *SMAD7* polymorphisms with CRC risk, because there were not sufficient studies conducted on other cancers.

To date, a large number of studies have focused on the relationship between *SMAD7* polymorphisms and cancer. However, controversies remain as whether those polymorphisms indeed associate with increased cancer risks. Our large-scale meta-analysis demonstrated that the C allele of rs4464148, the T allele of rs4939827, and the T allele of rs12953717 were all significantly associated with the increased CRC risk, which may provide a basis for genetic testing in the development of CRC. Consistent with our findings, Noci et al. [40] recently showed that *SMAD7* rs4939827 is also associated with cancer survival rate after therapy. Therefore, the identification of *SMAD7* polymorphisms may also benefit developing targeted and personalized therapy against CRC. However, more comparative studies are needed to evaluate interactions of *SMAD7* polymorphisms and cancer risk in other specific cancer subtypes and ethnic subtypes

## MATERIALS AND METHODS

### Literature Search strategy

We searched for relevant case-control studies using the following words and terms: “*SMAD7*”, “Mothers against decapentaplegic homolog 7”, “rs4464148”, “rs4939827”, “rs12953717”, “polymorphism” or “variation”, “susceptibility”, and “tumor” or “cancer” or “carcinoma” or “neoplasia” or “colorectal caner” or “CRC” in PubMed, the Web of Science, EBSCO, and Embase databases. There were no limitations on the language and year for the literature search. The last search was updated on June 30, 2016. References of the retrieved publications were also screened.

### Inclusion criteria

Two authors independently screened titles and abstracts to identify relevant studies. Full-text articles of these studies were then carefully read to select eligible studies. Studies had to meet the following inclusion criteria: (a) were a case-control study, nested case-control or a cohort study; (b) evaluated the association between *SMAD7* polymorphisms (“rs4464148”, “rs4939827”, and “rs12953717”) and CRC risk; (c) had available genotype frequencies both in cases and controls; (d) the genotype distribution in control groups was in Hardy-Weinberg equilibrium (HWE). (e) In cases of multiple studies with overlapping, redundant data published, only the most recent or complete study was included.

### Qualitative assessment

Two authors independently conducted the quality assessment. The Newcastle–Ottawa Scale (NOS) was used to evaluate the study quality, which scored studies by the selection of the groups, the comparability of cases and controls, and the ascertainment of the exposure. We considered a study awarded 0-3, 4-6, or 7-9 as a low-, moderate-, or high-quality study, respectively [23].

### Data extraction

Two authors independently selected the relevant articles and extracted the following data: first author's name, publication date, ethnicity, cancer type, genotyping methods, number of cases and controls, and number of genotypes in case-control groups. In addition, *P* values according to the HWE in controls were extracted from the included studies.

### Statistical analysis

Our meta-analysis was performed using Stata software (version 12.0; StataCorp LP, College Station, TX, USA). We first calculated the strength of the association between *SMAD7* polymorphisms and CRC by odds ratio (OR) corresponding to 95% confidence interval (CI) for different genetic models. Then we stratified the studies by ethnic subtypes and examined the association between *SMAD7* polymorphisms and the CRC risk (Table 2). A chi-square-based *Q*-statistic test [41] was performed to evaluate the between-study heterogeneity of the studies. *P_H_* < 0.05 was considered significant for heterogeneity. We also calculated the quantity *I^2^* that represents the percentage of total variation across studies. As a guide, values of *I^2^* less than 25% were considered “low”, values about 50% were considered “moderate”, and values greater than 75% were considered “high”[42]. The fixed effects model was used when there was no heterogeneity of the results of studies; otherwise, the random-effects model was chosen. A pooled OR obtained by meta-analysis was used to give a more reasonable evaluation of the association. The significance of the pooled OR was determined by *Z* test (*P_Z_* ≤0.05 suggests a significant OR). Funnel plots were used to access publication bias by the method of Begg's test and Egger's test. A *T* test was performed to determine the significance of the asymmetry. An asymmetric plot suggested possible publication bias (*P_E_* ≥ 0.05 suggests no bias).

## SUPPLEMENTARY FIGURES


