# Supplementary material for: SMAD7 polymorphisms and colorectal cancer risk: a meta-analysis of case-control studies
Source: Oncotarget. 2016 Sep 27;7(46):75561–70. doi: 10.18632/oncotarget.12285 (PMC5342761; doi:10.18632/oncotarget.12285)
Supplement: Supplementary file 1 [file oncotarget-07-75561-s001.pdf]

SMAD7 polymorphisms and colorectal cancer risk: a meta-analysis of case-control studies

SUPPLEMENTARY FIGURES

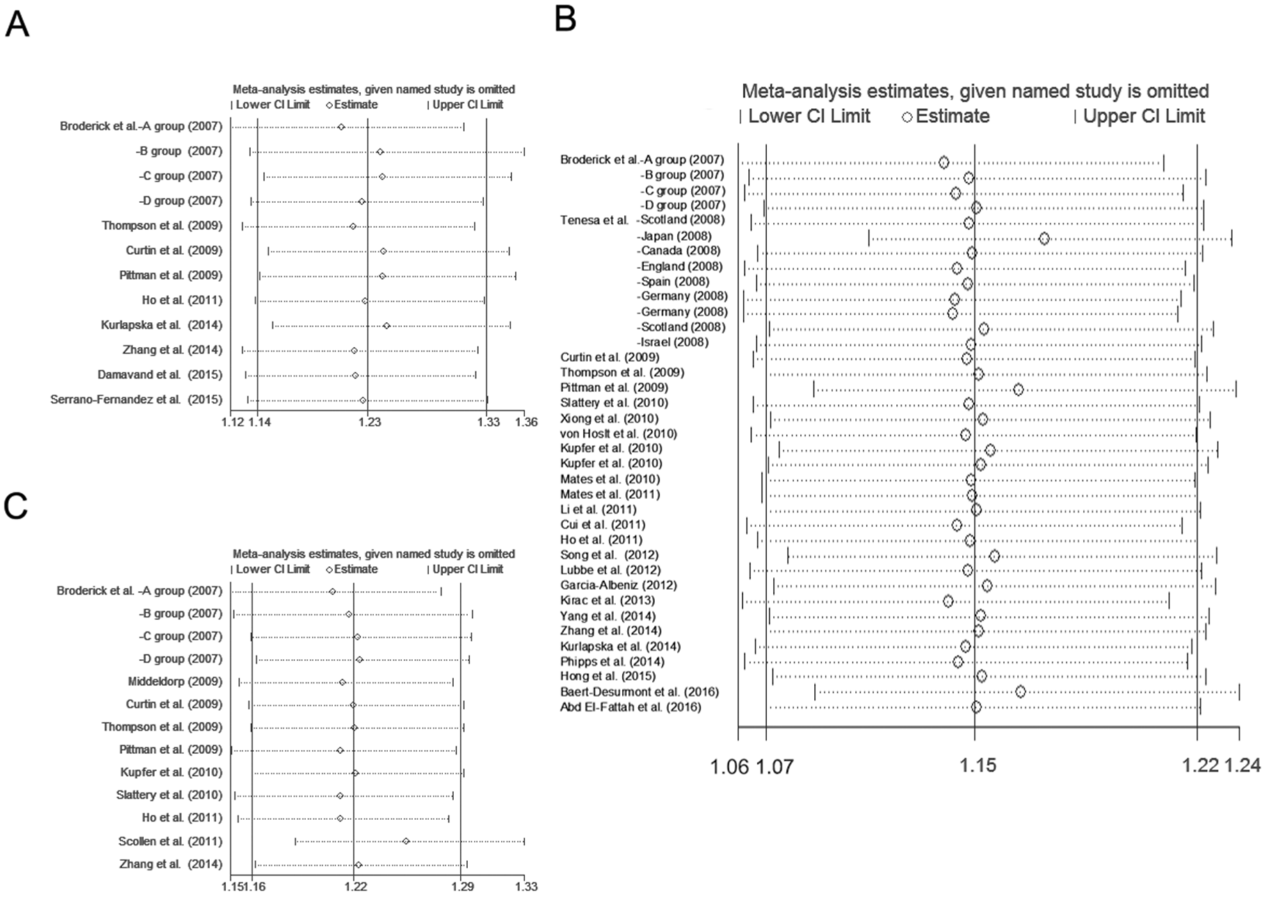

Supplementary Figure S1: One-way sensitivity analysis of the pooled ORs and 95% CI for *SMAD7* polymorphisms in a recessive model.

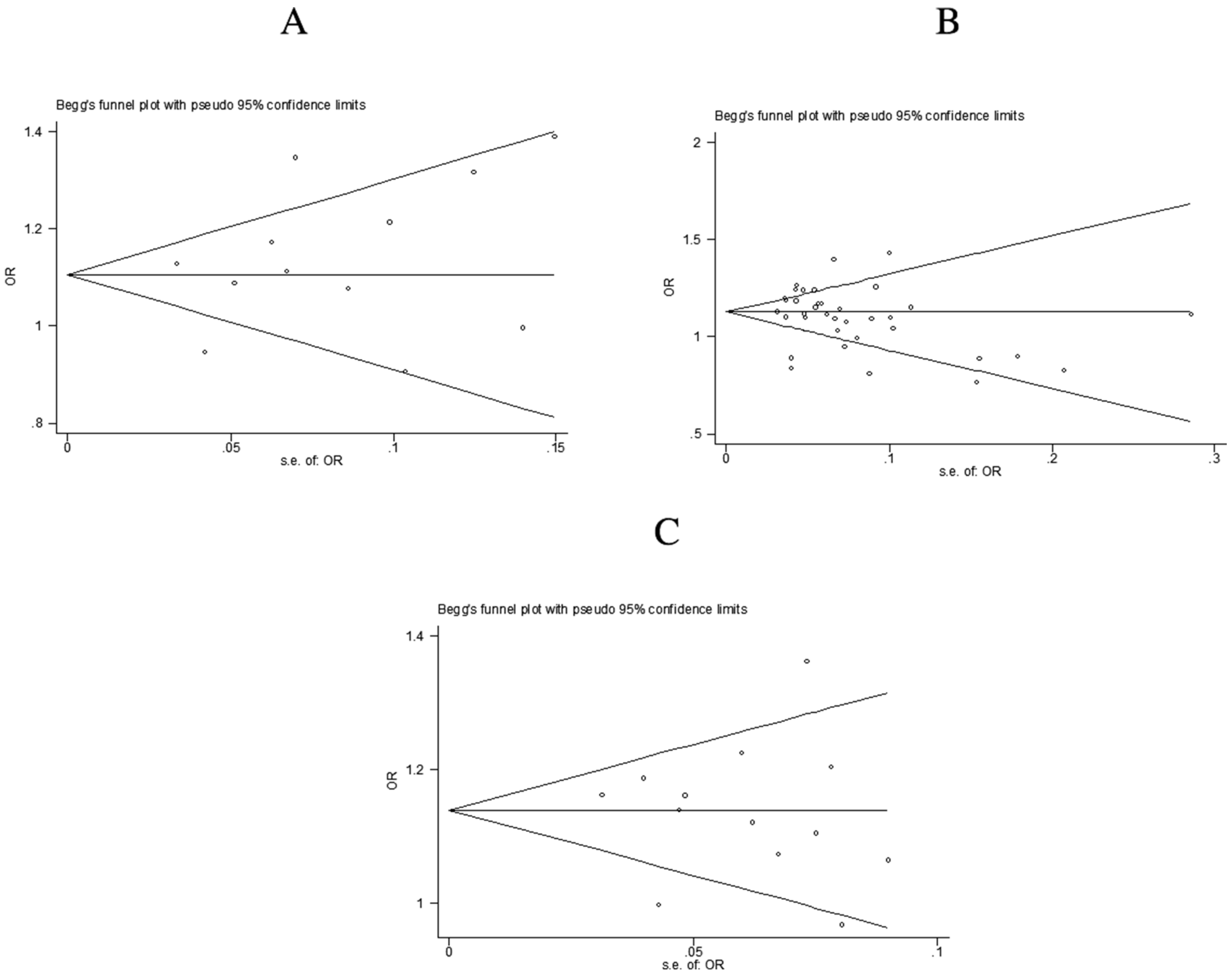

Supplementary Figure S2: Begg's funnel plot of the Egger's test for publication bias. A. In comparison of *SMAD7* rs4464148 allele C vs. allele T; **B.** *SMAD7* rs4939827 allele T vs. allele C; **C.** *SMAD7* rs12953717 polymorphism allele T vs. allele C.
